# Measurement of Serum Klotho in Systemic Sclerosis

**DOI:** 10.1155/2017/9545930

**Published:** 2017-08-22

**Authors:** Rossella Talotta, Sara Bongiovanni, Teresa Letizia, Federica Rigamonti, Maria Chiara Ditto, Fabiola Atzeni, Fausto Salaffi, Alberto Batticciotto, Maria Chiara Gerardi, Marco Antivalle, Tarcisio Vago, Maurizio Benucci, Piercarlo Sarzi-Puttini

**Affiliations:** ^1^Department of Rheumatology, University Hospital “Luigi Sacco”, Milan, Italy; ^2^Endocrinology and Rheumatology Laboratory, University Hospital “Luigi Sacco”, Milan, Italy; ^3^IRCCS Galeazzi Orthopedic Institute, Milan, Italy; ^4^Department of Rheumatology, Polytechnic University of the Marche, Ancona, Italy; ^5^Department of Rheumatology, “San Giovanni di Dio” Hospital, Florence, Italy

## Abstract

**Background:**

The aim of our study was to evaluate the serum concentration of klotho in a cohort of systemic sclerosis (SSc) patients compared to that of healthy controls and to correlate its levels with the degree and the kind of organ involvement.

**Methods:**

Blood samples obtained from both patients and controls were collected and analysed by an ELISA test for the determination of human soluble klotho. Scleroderma patients were evaluated for disease activity through clinical, laboratory, and instrumental assessment.

**Results:**

Our cohort consisted of 81 SSc patients (74 females, mean age 63.9 ± 13.1 years) and 136 healthy controls (78 females, mean age 50.5 ± 10.7 years). When matched for age, serum klotho concentration significantly differed between controls and patients (*p* < 0.001). However, in SSc patients, we did not find any significant association between serum klotho and clinical, laboratory, and instrumental findings. Lower serum levels of klotho were detected in 4 patients who were anticitrullinated peptide antibody (ACPA) positive (*p* = 0.005).

**Conclusions:**

Our data show a lower concentration of klotho in the serum of SSc patients compared to that of healthy controls, without any significant association with clinical manifestations and laboratory and instrumental findings. The association between serum klotho and ACPA positivity requires further investigation.

## 1. Introduction

Systemic sclerosis (SSc) is a connective tissue disease characterized by microangiopathy, activation of the immune system, and fibrosis. Raynaud's phenomenon, skin sclerosis, digital ulcers, subcutaneous calcinosis, lung interstitial disease, esophageal dysmotility, and renal crisis represent some of the most peculiar features of the disease. Endothelial dysfunction may represent the *primum movens* in the pathogenesis of SSc. Consecutive episodes of vasoconstriction and riperfusion contribute to the generation of oxidative stress, to the activation of the immune response, and to the induction of reparative processes, leading to fibrosis.

Klotho is a transmembrane and soluble protein that displays a glucosidase activity, although it mainly acts as a coreceptor. Klotho is involved in mineralization, electrolytic balance, insulin sensitivity, cell renewing, and reparative processes. Transgenic mice lacking klotho expression develop a phenotype characterized by accelerated aging, skin atrophy, lung emphysema, osteoporosis, delayed wound healing, and vascular calcification.

A deficiency in klotho expression could be responsible for microangiopathy, calcinosis, and fibrosis, which represent hallmarks of SSc.

We wondered, therefore, if serum concentration of klotho could be reduced in patients suffering from SSc, being also related to the severity of the disease or specific clinical manifestations.

The aim of our study was to evaluate the serum concentration of klotho in a group of SSc patients compared to healthy controls and to correlate its levels with the degree and the kind of organ involvement, assessed through Medsger's scale score and laboratory, instrumental, and functional tests.

## 2. Methods

We enrolled 81 consecutive Caucasian patients affected by SSc, according to 2013 ACR/EULAR criteria [1] and 136 matched healthy controls. Enrolment time took place since October 2014 until April 2016.

Blood samples obtained from both patients and controls were collected and analysed through an enzyme-linked immunosorbent assay (ELISA) assay for the determination of human soluble klotho by using a monoclonal anti-KL antibody and a KL-HRP conjugate (My Biosource, CA, USA). The sensitivity in this assay was 0.1 ng/mL. Blood samples were centrifuged, and sera were stored for a maximum of six months at −80°C until utilization.

Disease severity was scored through Medsger's scale [2]. In addition, modified Rodnan skin score (mRSS) for the degree and the extension of cutaneous sclerosis; pulmonary function tests for the assessment of forced vital capacity (FVC) and diffusing capacity or transfer factor of the lung for carbon monoxide per unit alveolar volume (DLCO/AV); chest high-resolution computed tomography (HRCT) scan for the detection of lung fibrosis; 2D echocardiography (2D-ECHO) for the screening of pulmonary artery hypertension (PAH); and laboratory tests for the determination of serum creatinine level, autoantibodies, erythrocyte sedimentation rate (ESR), and C-reactive protein (CRP) were also performed. Right heart catheterization was performed in a minority of patients but was not included in our analysis.

Subjects suffering from uncontrolled diabetes, lung obstructive disease, or renal failure unrelated to systemic sclerosis were excluded. Furthermore, patients with overlapping connective tissue diseases that could compromise the renal function, such as systemic lupus erythematosus (SLE), were also excluded from the analysis.

Data were reported as mean ± standard deviation (SD) or median, range, and interquartile range (IQR) as appropriate. Wilcoxon's test for unpaired samples was used for the comparison of klotho serum concentration between patients and healthy controls. In the group of scleroderma patients, Spearman's rank correlation coefficient was used to compare the level of klotho with clinical, laboratory, and instrumental findings. *p* value was set at <0.05. Statistical analysis was performed using a SPSS calculator, version 23.

The protocol was approved by our local ethic committee and registered with the number 88 of 05 February 2015 and conducted according to the Declaration of Helsinki. Patients and healthy subjects were informed about the procedures of the study and signed an informed consent.

## 3. Results

Our cohort consisted of 81 SSc patients (74 females, mean age 63.9 ± 13.1 years, mean disease duration 12.4 ± 8.9 years) and 136 healthy controls (78 females, mean age 50.5 ± 10.7 years). In the group of SSc patients, 22 (27.1%) suffered from diffuse form of disease and 59 (72.8%) from limited form. Median mRSS was 4.5 (range 0–30; IQR 6.8) and mean Medsger's scale score was 4.3 ± 2.3. Antinuclear antibodies (ANAs) were detected in 74 SSc patients (91.3%); among whom, 41 (50.6%) were positive for anticentromere and 19 (23.4%) for anti-SCL70 autoantibodies. Antiextractable nuclear antigen autoantibodies (ENA) anti-LaSSB and anti-RoSSA were present, respectively, in 2 and 10 patients; anticitrullinated peptide antibodies (ACPA) in 4 patients; rheumatoid factor (RF) in 1 patient; antimitochondrial antibodies (AMA) in 2 patients; antiphospholipid antibodies IgM and IgG in 6 patients; and anti-Jo1 and anti-ribonucleoprotein (RNP) autoantibodies, respectively, in 1 and 1 patient.

Chest HRCT showed pulmonary fibrosis in 35 (43.2%) patients; and 2D-ECHO detected the signs of PAH in 19 (23.4%) patients. Digital ulcers were objectified in 18 (22.2%) cases and calcinosis in 14 (17.3%) cases. Overall, laboratory tests showed normal to mild increased ESR values (median 15 mm 1st hour, range 2.0–84, IQR 22.8; normal range 0–20 mm), with a CRP within the range of normality (median 2.1 mg/L, range 0–24, IQR 3.6; normal range 0–10 mg/L). Renal function was globally preserved (median serum creatinine 0.7 mg/dL, range 0.49–2.22, IQR 0.3). Four patients showed an increase in serum creatinine value over the upper limit of the normal range without any further sign of kidney failure.

Table 1 shows the clinical and demographic characteristics of the SSc cohort.

Patients were treated with calcium channels blockers (27 cases), i.v. prostanoids (all), and immunosuppressive drugs (prednisone 2–12.5 mg/day in 26 cases; hydroxychloroquine 200–400 mg/day in 17 cases; mycophenolate mofetil 1-2 g/day in 3 cases; methotrexate 10–20 mg/week in 7 cases; cyclosporin A 100 mg/day in 1 case; and azathioprine 100–200 mg/day in 5 cases). Moreover, 23 patients assumed acetylsalicylic acid, 10 patients bosentan, and 14 patients angiotensin-converting enzyme inhibitors.

**Figure 1:**
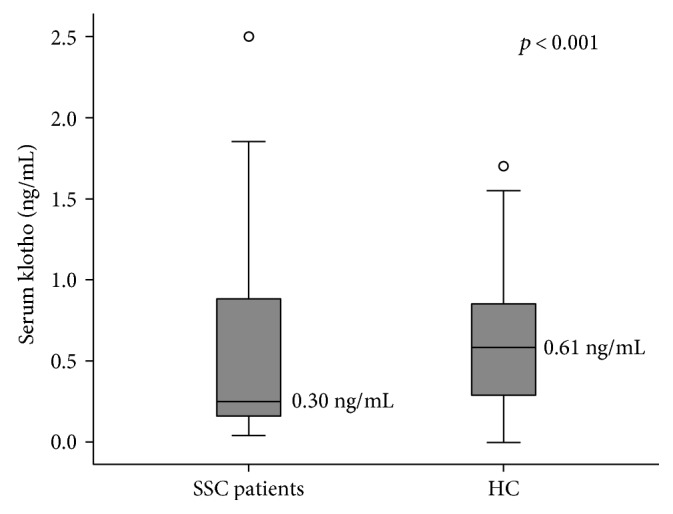
Serum klotho concentrations in matched scleroderma patients and healthy controls with reported median values. SSC = systemic sclerosis; HC = healthy controls.

In healthy controls, serum klotho concentration ranged from 0.0 to 1.70 ng/mL (median 0.61 ng/mL, IQR 0.60). In SSc patients, serum klotho concentration ranged from 0.0 to 2.50 ng/mL (median 0.30 ng/mL, IQR 0.79). These values are in line with previous results obtained from healthy population by means of ELISA assays [3, 4]. Serum klotho was not associated to sex or age in the group of scleroderma patients. On the contrary, in the control group, serum klotho was directly related to age (*ρ* 0.304, F 14.6, *p* < 0.001), despite that no association with sex was found. When the two groups were matched for age, serum klotho levels were significantly reduced in patients compared to controls (*p* < 0.001) (Figure 1).

**Figure 2:**
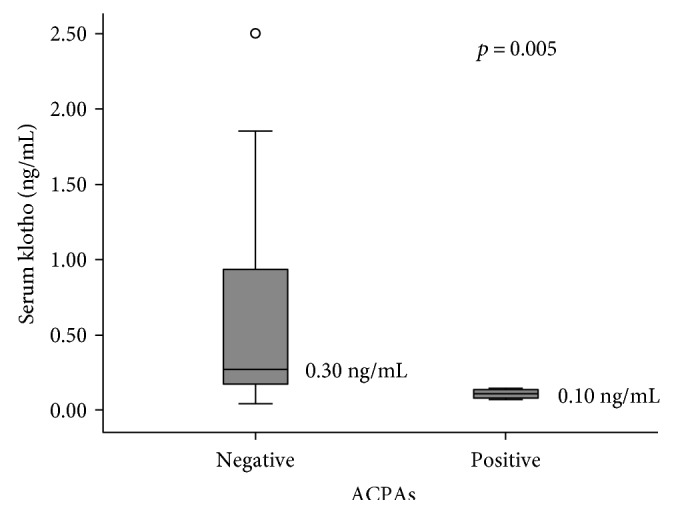
Serum klotho concentration with reported median values according to anticitrullinated peptide antibody (ACPA) positivity in scleroderma patients.

Spearman's test did not find any significant correlation between klotho and each of the clinical, laboratory, and instrumental variables. When patients were subdivided according to the presence of calcinosis, digital ulcers, lung fibrosis, echocardiographic signs of PAH, or disease subtype, no significant difference was observed in serum klotho concentration using a nonparametric test for unpaired samples (Wilcoxon's test). However, when evaluated for the presence of a specific pattern of autoantibodies, patients being ACPA positive showed significantly lower levels of serum klotho (median values 0.10 ng/mL versus 0.30 ng/mL; *U* 25.500, *Z* −2.802, *p* = 0.005) than those who were not (Figure 2). Serum klotho levels were not significantly influenced by concomitant treatments.

## 4. Discussion

Firstly described in 1997 by Kuro-o and colleagues as an aging-contrasting factor [5], klotho is a single-pass transmembrane protein, mainly expressed in the distal convolute tubules of the kidney and generated in 2 isoforms: *α*- and *β*-klotho. Klotho may act as a glucosidase or coreceptor by means of a paracrine or endocrine way [6]. Trasmembrane klotho cleaved by metalloproteinases in a soluble form maintains its functions [7]. This marker has gained interest over the time in the pathogenesis of kidney failure, where a lack of the protein seems to be associated to a declined estimated glomerular filtration rate [8–10]. More recently, klotho has been considered an antagonist for the proliferation of malignant cells, through fibroblast growth factor receptor (FGFR) and Wnt/*β*-catenin signalling pathways [11].

Soluble klotho may antagonize Wnt and transforming growth factor-*β* (TGF-*β*) signaling thus preventing tissue fibrosis and cancer metastasis [12–14].

It has been reported that klotho may increase the abundance on plasma membranes of transient receptor potential vanilloid receptor-related channels (TRPV5); it may interact with the Na^+^/K^+^ ATPase and insulin receptors and strengthen the affinity between fibroblast growth factors 19, 21, and 23 (FGF19, FGF21, and FGF23) and their receptors [15–17].

In particular, by interacting with FGF23 receptor, *α*-klotho may reduce the expression of *α*1-hydroxylase in the kidney, thus lowering the concentration of 1,25(OH)2D3 and preventing the reabsorption of calcium and phosphate from the kidney proximal tubules. Klotho mutant mice develop early hyperphosphatemia and hypercalcemia with a high risk of ectopic calcifications and accelerated osteoporosis due to a reduction in the number of both osteoclasts and osteoblasts.

Klotho is involved in the regulation of intracellular Ca^2+^ entry, whose amount presides over many biological activities, including vessel tone control and wound healing [18]. Calcium homeostasis is crucial for the correct functioning of endothelial cells. The amount of intracellular calcium, mainly released from endoplasmic reticulum, regulates many biological activities, including proliferation and apoptosis. TRP receptors modulate the entrance of calcium into endothelial cells in a klotho-dependent manner. In murine models, klotho seems in fact to prevent the junction disruption and the apoptosis of endothelial cells, by binding to the complex vascular endothelial growth factor receptor (VEGFR)/TRPC-1 and indirectly regulating the intracellular calcium concentration [19]. Endothelial cells from klotho mutant mice show a deficit in the internalization of TRPC-1 with an augmented calcium influx and a consequent increased activity of calcium-dependent proteases cleaving adhesion molecules. Moreover, a deficit in klotho expression is related to a reduced production of endothelial nitric oxide (NO), with a repercussion on vascular tone and apoptosis of vascular smooth muscle cells (VSMC).

Some authors have also demonstrated that klotho may inhibit the secretion of several cytokines, such as interleukin-6 and interleukin-8 (IL-6 and IL-8), by binding retinoic acid-inducible gene-I (RIG-1) [20]. Mice lacking klotho expression experiment an impairment in B-lymphopoiesis which is not rescued by the addiction of IL-7, while myeloid and erythroid cells are not affected [21].

Conversely, other studies have shown that other inflammatory cytokines, including tumor necrosis factor-*α* (TNF-*α*), may reduce klotho expression through NF-kB [22].

Systemic sclerosis is a connective tissue disease characterized by Raynaud's phenomenon and a progressive cutaneous fibrosis extending from distal to proximal sites. In the most severe forms, fibrosis affects also internal organs, determining various clinical pictures ranging from interstitial lung disease to restrictive heart disease or dysphagia, heavily influencing the prognosis. Although the disease is characterized by the detection of specific autoantibodies (mostly anticentromere and anti-SCL70), the pathogenic role of the immune system is probably less important than in other autoimmune connective tissue diseases. On the contrary, the endothelial dysfunction and the aberrant activation of fibroblasts play a more crucial role in the induction and maintenance of SSc. Several soluble vascular markers, including soluble intercellular adhesion molecule- (sICAM-) 1, vascular endothelial growth factor (VEGF), and endostatin, are altered in SSc and may vary according to the course of the disease [23, 24]. In addition, the research on animal models has demonstrated the involvement of endothelial cells, pericytes, and myofibroblasts in the pathogenesis of SSc, culminating in an impaired process of tissue healing and reparation [25].

**Figure 3:**
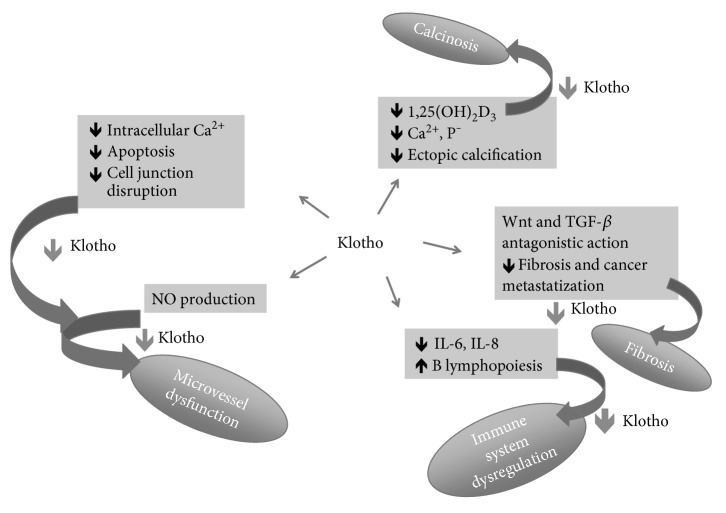
The possible pathway presided by klotho in the pathogenesis of systemic sclerosis. NO = nitric oxide; IL-6 = interleukin-6; IL-8 = interleukin-8; TGF-*β* = transforming growth factor-*β*, Ca^2+^ = calcium; P^−^ = phosphate.

Klotho participates to many biologic activities related to vascularization, collagen production, calcification, and tissue repair and may therefore be involved in the pathogenesis of SSc in several ways (Figure 3).

By interacting with VEGFR and TRP and regulating intracellular calcium balance, klotho may counteract the phenomenon of ectopic calcification, contribute to endothelial stability, and prevent oxidative stress [26]. Furthermore, klotho may inhibit the collagen deposition by antagonizing TGF-*β*. The secretion of some cytokines such as IL-6, which is fundamental for the maturation of plasma cells and the production of autoantibodies, may be increased in case of deficit of klotho. As demonstrated in several experiments on murine asthma models and human bronchial epithelial cells, klotho may counteract the remodeling of airways and prevent lung fibrosis by interfering with TGF-*β*1 and VEGF [27].

Klotho mutant mice display a phenotype characterized by accelerated aging, atherosclerosis, osteoporosis, and lung obstructive disease [5]. Moreover, mice lacking klotho expression have also a delay in skin wound healing, partly due to a deficit of type 1 and type 3 collagen depositions [18]. Similarly, SSc is currently considered as an accelerated aging phenotype, partly relying on altered epigenetic control [28, 29].

Our results show a significant difference in the serum concentration of klotho of SSc patients and healthy controls; however, the level of klotho was not associated to disease severity, according to Medsger's scale, and to clinical, laboratory, and instrumental findings, neither to a particular manifestation of the disease. In line with our data, a recent experiment investigating the association between klotho and precapillary PAH did not achieve any conclusive result [30]. We found a significant association between low klotho levels and the positivity for ACPAs. ACPAs represent a hallmark for rheumatoid arthritis (RA), and SSc patients having detectable ACPA titres often suffered from arthralgias or arthritis. A group of researchers demonstrated a reduced expression of klotho in CD4^+^ lymphocytes from RA patients [31]; however, it is unclear whether the reduced klotho expression could mirror an accelerating aging process of the cells belonging to the immune system.

The use of soluble klotho as a biomarker has been investigated in systemic lupus erythematosus (SLE) [32], and more recently, its proangiogenic effect has been tested in a wound healing assay using human SSc-derived microvascular endothelial cells [33].

Our pivotal study has some limits. Firstly, we did not analyse separately the serum concentration of alpha- and beta-klotho that may account for some differences in biological activities. Secondly, evidences showed that ELISA kits for klotho detection have low sensitivity, perhaps attributable to the recognition of different isoforms of the protein [3]. Moreover, our study evaluated the soluble fraction of klotho, exploring its hormonal functions; but we did not perform immunohistochemical tests on SSc tissues, so the real amount of klotho, either as soluble or transmembrane, produced in situ and presiding a paracrine control, was not assessed. Thirdly, the serum concentration of FGF23 was not measured, as well as of other endothelial or fibroblastic markers. Finally, we did not evaluate the presence of a receptorial resistance to klotho or the presence of dysfunctional isoforms of the molecule (e.g., polymorphic variants).

## 5. Conclusions

Klotho is a novel biomarker involved in aging, cancer, and multiorgan dysfunction. Among its biological activities, klotho preserves the integrity of microvessels and favors the process of healing and tissue reparation. A deficit in klotho concentration may explain some of the clinical manifestation observed in SSc, such as digital ulcers, calcinosis, or fibrosis. Our data show a significant difference in the concentration of serum klotho between SSc patients and controls with an overall serum concentration being in line with that of general population. Nevertheless, we did not find any significant association among the serum levels of klotho and clinical manifestations of the disease or the severity of SSc expression. We found a significant association between low serum klotho concentration and the presence of ACPAs, but the significance of this finding is unclear. In conclusion, as this was a pivotal study and some limits can occur, further studies in order to understand the real involvement of klotho in the pathogenesis of SSc are suggested.

## Figures and Tables

**Table 1:** Demographic and clinical characteristics of SSc patients.

Characteristics	Scleroderma patients (number 81)
Age (years) (mean ± standard deviation)	63.9 ± 13.1
Sex (F/M) (number)	74/7
Disease duration (years) (mean ± standard deviation)	12.4 ± 8.9
Clinical form (limited/diffuse) (number)	59/22
Antinucleus autoantibody positivity (number)	74
Anticentromere autoantibody positivity (number)	41
Anti-SCL70 autoantibody positivity (number)	19
Erythrocyte sedimentation ratio, 1st hour mm (median, range)	15, 2.0–84
C-reactive protein, mg/L (median, range)	2.1, 0–24
Serum creatinine, mg/dL (median, range)	0.7, 0.49–2.22
Digital ulcers (number)	18
Calcinosis (number)	14
Pulmonary artery hypertension screened through 2D echocardiography (number)	19
Lung interstitial disease (number)	35
Diffusing capacity or transfer factor of the lung for carbon monoxide per unit alveolar volume (%) (mean ± standard deviation)	83.3 ± 15.2

Vital capacity (%) (mean ± standard deviation)	100.3 ± 17.4
Medsger's scale score (mean ± standard deviation)	4.3 ± 2.3
Modified Rodnan skin score (median, range)	4.5, 0–30
Osteoporosis (number)	32

## Abbreviations

SSc: Systemic sclerosis
ACPAs: Anticitrullinated peptide antibodies
FGFR: Fibroblast growth factor receptor
SLE: Systemic lupus erythematosus
mRSS: Modified Rodnan skin score
FVC: Forced vital capacity
DLCO/AV: Diffusing capacity or transfer factor of the lung for carbon monoxide per unit alveolar volume
HRCT: High-resolution computed tomography
2D-ECHO: 2-dimensional Echocardiography
PAH: Pulmonary artery hypertension
ESR: Erythrocyte sedimentation rate
CRP: C-reactive protein
SD: Standard deviation
IQR: Interquartile range
ANAs: Antinuclear antibodies
ENA: Antiextractable nuclear antigen antibodies
AMA: Antimitochondrial antibodies
RNP: Ribonucleoprotein
TRPV5: Transient receptor potential vanilloid receptor-related channels
VEGFR: Vascular endothelial growth factor receptor
NO: Nitric oxide
VSMC: Vascular smooth muscle cells
RIG-1: Retinoic acid-inducible gene-I
IL: Interleukin
TNF-*α*: Tumor necrosis factor-*α*
TGF-*β*: Transforming growth factor-*β*
sICAM-1: Soluble intercellular adhesion molecule-1
VEGF: Vascular endothelial growth factor
RA: Rheumatoid arthritis.

## Additional Points

Datasets are recorded and available at the Department of Rheumatology of the University Hospital “Luigi Sacco,” Milan. Blood samples were collected and stored at the laboratories of the same department.
